# Supplementary material for: Nasal Immunization with the C-Terminal Domain of Bcla3 Induced Specific IgG Production and Attenuated Disease Symptoms in Mice Infected with Clostridioides difficile Spores
Source: Int J Mol Sci. 2020 Sep 13;21(18):6696. doi: 10.3390/ijms21186696 (PMC7555657; doi:10.3390/ijms21186696)
Supplement: Supplementary file 1 [file ijms-21-06696-s001.pdf]

Table S1. Highly immunogenic peptides for MHC-I molecules on the C-terminal domain of BclA3.

Allele [a]	Start [b]	End [b]	Percentile Rank [c]
HLA-B*44:03	646	654	0.11
HLA-A*26:01	571	579	0.12
HLA-A*68:01	561	569	0.12
HLA-A*26:01	591	600	0.12
HLA-B*15:01	600	609	0.13
HLA-B*51:01	567	576	0.14
HLA-B*07:02	567	576	0.17
HLA-A*33:01	561	569	0.18
HLA-B*07:02	511	519	0.2
HLA-A*68:02	609	617	0.2
HLA-A*26:01	662	670	0.21
HLA-A*68:01	560	569	0.21
HLA-A*11:01	560	569	0.22
HLA-A*68:01	641	649	0.23
HLA-A*68:02	666	675	0.27
HLA-B*07:02	604	612	0.3
HLA-A*30:02	591	600	0.3
HLA-A*31:01	560	569	0.33
HLA-A*02:03	527	535	0.33
HLA-A*02:06	580	589	0.34
HLA-A*01:01	591	600	0.35
HLA-B*53:01	567	576	0.35
HLA-A*02:03	618	626	0.38
HLA-A*30:02	571	579	0.38
HLA-A*02:01	650	658	0.4
HLA-B*07:02	108	116	0.4
HLA-B*44:03	646	655	0.41
HLA-B*44:02	646	654	0.42
HLA-A*02:03	656	664	0.43
HLA-B*40:01	646	654	0.46
HLA-A*26:01	591	599	0.47
HLA-A*30:02	662	670	0.47
HLA-B*53:01	513	521	0.5
HLA-B*15:01	662	670	0.5
HLA-A*68:02	581	589	0.5
HLA-A*68:02	591	599	0.5
HLA-B*58:01	642	650	0.5
HLA-B*35:01	511	519	0.5
HLA-A*30:02	661	670	0.53
HLA-A*33:01	560	569	0.53
HLA-A*31:01	561	569	0.55
HLA-A*23:01	578	586	0.56
HLA-A*68:02	580	589	0.56
HLA-B*51:01	517	525	0.6
HLA-A*02:03	650	658	0.66
HLA-A*02:06	644	653	0.67
HLA-B*57:01	642	650	0.68
HLA-A*11:01	591	600	0.69

HLA-B*51:01	511	519	0.7
HLA-B*15:01	571	579	0.7
HLA-A*68:02	628	636	0.7
HLA-A*02:03	580	589	0.72
HLA-A*02:03	662	671	0.73
HLA-A*03:01	560	569	0.74
HLA-A*01:01	661	670	0.8
HLA-B*35:01	567	575	0.8
HLA-A*68:02	582	591	0.83
HLA-A*26:01	572	580	0.84
HLA-A*24:02	578	586	0.9
HLA-A*68:02	654	662	0.9
HLA-B*07:02	567	575	0.92
HLA-A*02:06	656	664	0.95
HLA-A*68:02	654	663	0.97

a—The choice of alleles was based on Human flequently occurring alleles and on the reference set of alleles recomended by Immune Epitope Data Base (IEDB). b—Position of the immunogenic epitope on BclA3 amino acid sequence. c - Epitopes with Percentile Rank under 1 are considered highly immunogenic by default according to prediction methods used recommended by IEDB. It was found 63 immunogenic peptides with Percentile Rank under 1 on the C—terminal domain of BclA3.

Table S2. Highly immunogenic peptides for MHC-I molecules on the N-terminal domain of BclA3.

Allele [a]	Start [b]	End [b]	Percentile Rank [c]
HLA-B*07:02	53	62	0.19
HLA-B*07:02	50	58	0.2
HLA-B*07:02	53	61	0.2
HLA-B*07:02	108	117	0.51
HLA-B*07:02	500	509	0.51
HLA-B*07:02	50	59	0.52
HLA-B*07:02	62	71	0.52
HLA-B*07:02	175	184	0.53
HLA-B*07:02	363	372	0.56
HLA-B*07:02	125	133	0.8
HLA-B*07:02	128	136	0.8
HLA-B*07:02	143	151	0.8
HLA-B*07:02	158	166	0.8
HLA-B*07:02	172	180	0.8
HLA-B*07:02	218	226	0.8
HLA-B*07:02	236	244	0.8
HLA-B*07:02	251	259	0.8
HLA-B*07:02	265	273	0.8
HLA-B*07:02	285	293	0.8
HLA-B*07:02	345	353	0.8
HLA-B*07:02	360	368	0.8
HLA-B*07:02	392	400	0.8
HLA-B*07:02	407	415	0.8
HLA-B*07:02	440	448	0.8
HLA-B*07:02	488	496	0.8
HLA-B*07:02	59	67	0.9
HLA-B*07:02	75	83	0.9
HLA-B*07:02	78	86	0.9
HLA-B*07:02	122	130	0.9

HLA-B*07:02	425	433	0.9
HLA-B*07:02	458	466	0.9

a—The choice of alleles was based on Human flequently occurring alleles and on the reference set of alleles recomended by Immune Epitope Data Base (IEDB). b—Position of the immunogenic epitope on BclA3 amino acid sequence. c—Epitopes with Percentile Rank under 1 are considered highly immunogenic by default according to prediction methods used recommended by IEDB. It was found 30 immunogenic peptides with Percentile Rank under 1 on the N- terminal domain of BclA3.

Table S3. Highly immunogenic peptides for MHC-II molecules on the C-terminal domain of BclA3.

Allele [a]	Start [b]	End [b]	Percentile Rank [c]
HLA-DRB1*09:01	603	617	0.01
HLA-DRB1*09:01	604	618	0.01
HLA-DRB1*09:01	605	619	0.01
HLA-DRB1*09:01	606	620	0.01
HLA-DRB1*09:01	607	621	0.01
HLA-DPA1*01:03/DPB1*02:01	639	653	0.01
HLA-DPA1*01:03/DPB1*02:01	640	654	0.01
HLA-DPA1*01:03/DPB1*02:01	641	655	0.01
HLA-DPA1*01:03/DPB1*02:01	642	656	0.01
HLA-DPA1*01:03/DPB1*02:01	643	657	0.01
HLA-DRB1*07:01	574	588	0.01
HLA-DRB1*07:01	575	589	0.01
HLA-DRB1*07:01	576	590	0.01
HLA-DRB1*07:01	577	591	0.01
HLA-DRB1*07:01	578	592	0.01
HLA-DPA1*01/DPB1*04:01	643	657	0.01
HLA-DRB1*07:01	579	593	0.03
HLA-DPA1*01/DPB1*04:01	644	658	0.03
HLA-DPA1*01/DPB1*04:01	645	659	0.04
HLA-DPA1*01:03/DPB1*02:01	644	658	0.06
HLA-DRB1*01:01	606	620	0.09
HLA-DRB1*09:01	532	546	0.1
HLA-DRB1*09:01	533	547	0.1
HLA-DRB1*09:01	534	548	0.1
HLA-DRB1*09:01	535	549	0.1
HLA-DRB1*09:01	574	588	0.12
HLA-DRB1*09:01	608	622	0.13
HLA-DRB1*09:01	609	623	0.13
HLA-DRB1*09:01	575	589	0.16
HLA-DRB1*09:01	576	590	0.19
HLA-DRB1*01:01	607	621	0.19
HLA-DRB1*09:01	510	524	0.23
HLA-DPA1*01:03/DPB1*02:01	575	589	0.23
HLA-DPA1*01:03/DPB1*02:01	574	588	0.24
HLA-DPA1*01:03/DPB1*02:01	576	590	0.24
HLA-DPA1*01:03/DPB1*02:01	577	591	0.24
HLA-DRB1*09:01	509	523	0.28
HLA-DRB1*01:01	605	619	0.32
HLA-DRB1*01:01	608	622	0.32
HLA-DPA1*01:03/DPB1*02:01	578	592	0.34
HLA-DRB1*09:01	511	525	0.38
HLA-DRB1*09:01	508	522	0.44

HLA-DRB1*09:01	573	587	0.45
HLA-DRB1*09:01	577	591	0.47
HLA-DRB1*04:01	646	660	0.48
HLA-DRB1*04:01	647	661	0.48
HLA-DRB1*04:01	648	662	0.48
HLA-DRB1*04:01	649	663	0.48
HLA-DRB1*08:02	648	662	0.49
HLA-DRB1*08:02	571	585	0.5
HLA-DRB1*04:01	573	587	0.52
HLA-DRB1*11:01	644	658	0.55
HLA-DRB1*11:01	645	659	0.55
HLA-DRB1*11:01	646	660	0.55
HLA-DRB1*11:01	647	661	0.55
HLA-DRB1*11:01	648	662	0.55
HLA-DRB1*11:01	649	663	0.55
HLA-DRB1*11:01	650	664	0.55
HLA-DRB1*08:02	649	663	0.57
HLA-DRB1*04:01	650	664	0.57
HLA-DQA1*01:02/DQB1*06:02	634	648	0.6
HLA-DRB1*08:02	570	584	0.61
HLA-DRB1*04:01	574	588	0.67
HLA-DRB1*08:02	647	661	0.69
HLA-DRB1*08:02	646	660	0.7
HLA-DRB1*09:01	507	521	0.71
HLA-DRB1*08:02	572	586	0.71
HLA-DRB1*01:01	647	661	0.71
HLA-DQA1*01:02/DQB1*06:02	633	647	0.73
HLA-DRB1*08:02	645	659	0.73
HLA-DRB1*08:02	660	674	0.74
HLA-DRB1*04:05	647	661	0.75
HLA-DRB1*04:05	648	662	0.76
HLA-DPA1*02:01/DPB1*14:01	575	589	0.76
HLA-DRB1*04:05	645	659	0.79
HLA-DRB1*04:05	646	660	0.8
HLA-DRB1*04:05	644	658	0.81
HLA-DRB1*09:01	578	592	0.82
HLA-DQA1*01:02/DQB1*06:02	635	649	0.82
HLA-DRB1*04:01	575	589	0.85
HLA-DRB1*04:01	644	658	0.87
HLA-DRB1*04:01	645	659	0.87
HLA-DRB1*01:01	650	664	0.88
HLA-DRB1*04:01	596	610	0.88
HLA-DRB1*04:01	595	609	0.89
HLA-DPA1*02:01/DPB1*14:01	576	590	0.89
HLA-DRB1*04:01	594	608	0.9
HLA-DRB1*09:01	572	586	0.93
HLA-DRB1*08:02	606	620	0.95
HLA-DRB1*01:01	603	617	0.96
HLA-DRB1*01:01	604	618	0.96
HLA-DRB1*04:01	597	611	0.97

a—The choice of alleles was based on Human flequently occurring alleles and on the reference set of alleles recomended by Immune Epitope Data Base (IEDB). b—Position of the immunogenic epitope on BclA3 amino

acid sequence. c—Epitopes with Percentile Rank under 1 are considered highly immunogenic by default according to prediction methods used recommended by IEDB. It was found 91 immunogenic peptides with Percentile Rank under 1 on the C- terminal domain of BclA3. N-terminal domain and central region of BclA3 have not displayed peptides with Percentile Rank under 1 for MHC-II.
